# Supplementary material for: Vaccination against HBV and HAV as Mode of Hepatitis Prevention among People Living with HIV—Data from ECEE Network Group
Source: Vaccines (Basel). 2023 May 14;11(5):980. doi: 10.3390/vaccines11050980 (PMC10222000; doi:10.3390/vaccines11050980)
Supplement: Supplementary file 1 [file vaccines-11-00980-s001.zip › vaccines-2308398-supplementary.pdf]

Table S1. Survey content.

Question	Answer
What is your country	Text
What is the name of your clinic	Text
Your e-mail	Text
Your name and surname	Text
Does your clinic screen all HIV positive patients for HCV antibodies?	Yes<br>No
How frequently do you screen HIV positive patients for HCV antibodies?	New patients for the first time<br>Once a year<br>Patients with unexplained increase of hepatic transaminases<br>Patients with risky behaviour<br>Women who plan pregnancy or are pregnant<br>Other (please specify)
Is HCV testing free of charge in your country?	Yes, health insurance<br>Yes, other governmental programs<br>Yes, NGOs<br>No, but available as paid service for the patients<br>No, not available at all
Does your clinic screen all HIV positive patients for HBsAg?	Yes<br>No
How frequently do you screen HIV positive patients for HBsAg?	New patients<br>Once a year<br>Patients with unexplained increase of hepatic transaminases<br>Women who plan pregnancy or are pregnant<br>Other (please specify)
Is HBV testing free of charge in your country?	Yes, health insurance<br>Yes, other governmental programs<br>Yes, NGOs<br>No, but available as paid service for the patients<br>No, not available at all
Does your clinic screen all HIV positive patients for HAV IgG antibodies?	Yes<br>No
Do you screen all HBsAg positive persons for hepatitis Delta in your clinic?	Yes<br>No
Do you vaccinate all HIV positive patients against HAV?	Yes<br>No
Are vaccinations against HAV free of charge in your country?	Yes<br>No
If not, what is the average price (in EURO) of HAV vaccination?	Number
Do you vaccinate all HIV positive patients against HBV?	Yes<br>No
Are vaccinations against HBV free of charge in your country?	Yes<br>No

If not, what is the average price (in EURO) of HBV vaccination?	Number
Do you have your own national recommendations regarding vaccinations against HAV and HBV?	Yes<br>No
If not, which recommendations does your clinic use?	EACS recommendations<br>WHO guidelines<br>Other (please specify)
Does your clinic have a protocol for reviewing vaccine response (Hep A and B Ab titres) and re-vaccinating where needed?	Yes<br>No
Which NRTI does your clinic start in people with anti-HBs<10 IU/L post vaccination?	TDF/FTC<br>TDF/3TC<br>TAF/FTC<br>ABC/3TC<br>AZT/3TC<br>Other (please specify)
Which NRTI does your clinic start in people with chronic hepatitis B (HBsAg positive> 6 months)?	TDF/FTC<br>TDF/3TC<br>TAF/FTC<br>ABC/3TC<br>AZT/3TC<br>Other (please specify)
Do you have an access to DAA drugs in your country?	Yes<br>No
If yes, do you have any limitations for DAA public treatment access in your country (e.g., Not available for active IDU, alcohol abuse, migrants even if insured, prisoners)?	Yes<br>No<br>If yes, what kind of limitations
Is acute hepatitis C treatment available in your country?	Yes<br>No
If yes, is acute hepatitis C treatment reimbursed in your country?	Yes<br>No
When HCV - infected patients can be started to treat at the earliest in your country?	8 weeks<br>12 weeks<br>6 months<br>Other (please specify)

Note: NGO—non-governmental organization; HBV—Hepatitis B virus; HBsAg—Hepatitis B surface antigen; HCV—Hepatitis C virus: HAV—Hepatitis A virus; EACS—European AIDS Clinical Society; WHO—World Health Organization; DAA—direct-acting antiviral; NRTI—Nucleoside reverse transcriptase inhibitors; TDF—tenofovir; FTC—emtricitabine; 3TC—lamivudine; ABC—abacavir; AZT—zidovudine; TAF— tenofovir alafenamide; IDU—intravenous drug use.
